# Bibliographical

**Published:** 1897-09

**Authors:** 


### Bibliographical.

"The American Text Book of Operative Dentistry," published by Lea Bros. & Co. of Philadelphia, Pa., and edited by Edward C. Kirk, D.D.S., has just come to hand. It is the production of a number of authors under the editorial direction of Dr. Kirk.

A more extended notice of this work will appear in the near future in the REGISTER.

